# Assessment of computer vision syndrome and associated factors among employees of Ethio-telecom in Addis Ababa, Ethiopia

**DOI:** 10.3389/fpubh.2025.1524173

**Published:** 2025-02-05

**Authors:** Natnael Gizachew, Teferi Abegaz, Tenaw Demis, Melese Gashaw, Lidetu Demoze

**Affiliations:** ^1^School of Public Health, College of Health Science and Medicine, Dilla University, Dilla, Ethiopia; ^2^School of Public Health, College of Health Sciences, Addis Ababa University, Addis Ababa, Ethiopia; ^3^Federal Democratic Republic of Ethiopia Ministry of Labour and Skills, Addis Ababa, Ethiopia; ^4^Department of Environmental and Occupational Health and Safety, Institute of Public Health, College of Medicine and Health Sciences, University of Gondar, Gondar, Ethiopia

**Keywords:** computer vision syndrome, prevalence, determinants, Ethio telecom workers, task illumination, viewing distance, Addis Ababa, Ethiopia

## Abstract

**Background:**

Computer vision syndrome refers to a range of eye and vision-related problems which may result from extended use of digital devices such as computers. It is a public health problem, affecting more than 70% of all computer users. In developing countries like Ethiopia, there is scarcity of studies on computer vision syndrome, particularly in the telecom industry making evidence-based interventions difficult. Hence, the study aims to identify the determinants and the prevalence of computer vision syndrome among Ethio telecom workers in Addis Ababa, Ethiopia.

**Objectives:**

This study aimed to determine the prevalence of CVS and identify its factors among Ethio telecom workers in Addis Ababa, Ethiopia.

**Methods:**

Institution-based cross-sectional study was conducted among Ethio telecom employees in Addis Ababa, Ethiopia from March to June 2023. A total of 497 individuals participated in the study. Data were collected using an interviewer- administered questionnaire through Kobo Toolbox, observational checklist and an illuminance measuring instrument. The data were transferred to Statistical Package for the Social Sciences (SPSS) version 26 for analysis. Multivariable logistic regression was performed to assess the association and control for potential confounders.

**Results:**

The prevalence of CVS among Ethio telecom workers was 68.8% (95% CI: 64.5–72.9). Significant associations were observed with viewing distance <50 cm (AOR: 2.32, 95% CI: 1.24–4.33), improper task illumination (AOR: 1.78, 95% CI: 1.09–2.91), habit of taking breaks (AOR: 0.439, 95% CI: 0.281–0.686), and adjustment of brightness and contrast (AOR: 0.39, 95% CI: 0.22–0.68).

**Conclusion:**

More than two-thirds of Ethio telecom workers in Addis Ababa suffer from CVS, with significant influences from viewing distance, task illumination, breaks, and monitor settings. These findings underscore the need for interventions to enhance working conditions and reduce CVS prevalence among computer users.

## 1 Introduction

Technological advances have made a huge impact on almost every aspect of our lives. In particular, the availability of computers has simplified many daily tasks (1–3). The use of digital devices has increased substantially in recent years across all age groups. The extensive daily use for both social and professional purposes has become routine (4). Computers are one of the commonest office tools in various institutions such as government offices and their use has become essential worldwide (5). As a result, workers spend more time looking at computers and other digital devices. However, prolonged use of computers for extended hours causes a range of impairments such as vision-related problems, headaches, and low backache (6).

Computer vision syndrome has been described in various literature sources. The American Optometric Association provides the most widely used definition, stating that “CVS is a group of eye and vision-related problems resulting from prolonged use of computers, tablets, and cell phones” (1). It is a form of repetitive strain disorder that frequently occurs among people using visual display terminals such as computers, tablets, and cell phones for a prolonged duration (7). The level of discomfort has been seen to be proportional to the intensity of computer use (8).

Computer Vision Syndrome has become a growing public health concern often stated the 21st-century’s most common occupational hazard affecting more than 70% of all computer users and it is estimated that around 60 million people worldwide are affected by the problem with a million new cases occurring each year (9, 10) resulting in decreased workplace productivity, lower job satisfaction, higher error rates, and impaired visual ability (11–13). High workload, inadequate accessibility, low utilization of personal protective equipment, and restricted break times when using a computer make the problem of CVS even worse in low-income countries.

While there is no strong evidence for causation, studies have suggested that multiple factors may contribute to the development of this condition. These factors include prolonged exposure to computer screens, environmental factors, and workplace ergonomics (14, 15). The most commonly identified factors that play a role in the development of CVS in different pieces of literature are: Duration of computer use, pre-existing eye illness, knowledge about adverse health effects of prolonged computer use, rest break habits, brightness adjustment, sitting position, and age were significant factors affecting CVS in different pieces of literature (6–8).

Proper lighting is an important factor for visual efficiency and comfort. Consequently, improper lighting is often considered the biggest environmental factor that contributes to visual discomfort (16). There is a paucity of studies evaluating the influence of lighting conditions in the workplace on visual health. Therefore, this study aims to explore the effects of lighting and other ergonomic conditions to improve our understanding of their impact within this context. It attempts to close the gap in the literature by taking measurements such as lighting levels at workstations, and visual distance from monitors to account for environmental and ergonomic factors.

## 2 Materials and methods

### 2.1 Study area

**Figure 1:**
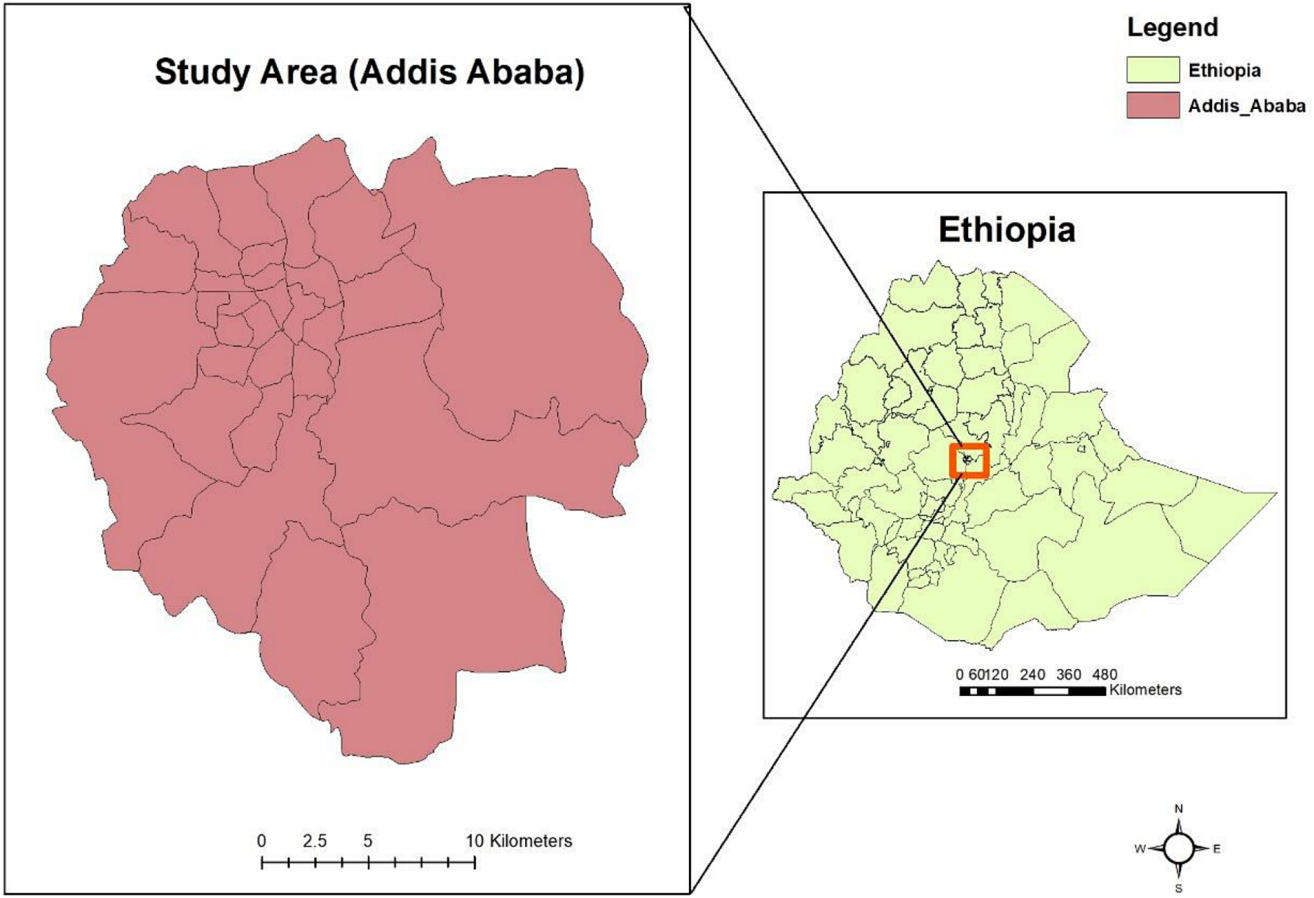
Study area map (Addis Ababa, Ethiopia).

The study was conducted in Ethio Telecom Zonal office branches in Addis Ababa, Ethiopia. Addis Ababa, the capital and the most important commercial and cultural center of Ethiopia, is geographically located at the heart of the nation (Figure 1). As of October 2022, Ethio Telecom has become the 23rd largest operator in the world from 781 operators and the 2nd largest operator in Africa from 198 operators. As of the time mentioned Ethio Telecom has 68.3 million subscribers. Its telecom service coverage and density have also reached 99.1% of the population, 85.4% of the Geography, and 64.5% of tele density, respectively, (17). Until recently, Ethio Telecom had a monopoly on all telecom services in Ethiopia. It employs approximately 12,288 individuals across the country.

### 2.2 Study design, sample size determination, and sampling techniques

An institution-based cross-sectional study was conducted from April to June 2023 to assess the prevalence and associated factors of CVS among computer-using employees of Ethio Telecom. All Ethio telecom employees who use computers in Addis Ababa were considered as the source population. Among them: Selected employees who use computers in the 6 Zonal district offices of Ethio Telecom across Addis Ababa were the study population.

#### 2.2.1 Inclusion criteria

Workers who used computers for at least 2 h daily in the past 12 months were eligible for the study (5).

#### 2.2.2 Exclusion criteria

Individuals on leave during the duration of the data collection period (18).

The sample size for the prevalence of CVS among Ethio-telecom employees was determined using a single population proportion formula with assumptions of the level of significance (*α*) = 5%, marginal error *d* = 4%, and prevalence of 74.6% (6) from a study on CVS among Employees of the Commercial Bank of Ethiopia in Addis Ababa, Ethiopia.


$$n=\frac{{\left(Z\alpha /2\right)}^{2}p\left(1-p\right)}{d^{2}}$$

After adding a 10% non-response rate the final sample size was 506.

From the total of 2,632 worker across the 6 zonal offices, Simple random sampling with proportional allocation was implemented in this study to reach sample participants.

### 2.3 Data collection tools and techniques

A structured questionnaire, adapted from similar literature, was used to collect data. The questionnaire was initially prepared in English, then translated into Amharic, and subsequently retranslated into English by experts to ensure consistency. A brief explanation about the purpose of the study was provided to the participants before conducting face-to-face interviews. The questionnaire included sections to collect data on background variables, symptoms of CVS, and personal characteristics questions related to CVS.

Observational Checklist: an observational checklist developed from a review of the literature was used to assess environmental and ergonomic-related conditions.

Measuring viewing distance: a measuring tape was used to measure the viewing distance between the top of the monitor and the level of the participant’s face.

Measuring task illumination levels: task illumination levels were measured using an instrument lux meter. A total of four measurements were taken at the task area (computer workstation): two on the top of the monitor screen 10 cm apart and two measurements were also taken 20 cm apart from the keyboard positions (17). The measurements were averaged to obtain the average illumination level of the task area.

The data collection process involved three environmental health professionals as data collectors and one supervisor overseeing the procedure. Participants’ responses were recorded digitally on mobile phones using the Kobo Toolbox platform. Data collection was completed over 5 weeks, from April 19 to May 24.

### 2.4 Operational definition

Computer Vision Syndrome (CVS): The presence of at least one symptom, either intermittently or continuously, for at least 1 week, during the last 12 months in the eyes was considered as the presence of CVS (6). Symptoms considered are blurred vision, eye strain, eye fatigue, redness of eyes, watery eyes, eye dryness, double vision, eye irritation, burning sensation, and headache.

Distance from monitor: The distance from the face to the monitor is measured in centimeters. A measurements ≥50 cm was considered proper viewing distance, while a measurement <50 cm was considered improper viewing distance (13).

Task illumination: the average amount of light falling on the task surface measured at the height of the task, an average measurement of ≥200 LUX at the task area is deemed appropriate lighting level. An average measurement below 200 LUX was considered inappropriate lighting (19, 20).

Adjustment of computer brightness: Adjusting the brightness of the computer so it is balanced with surrounding light.

Rest breaks: taking breaks for at least 15 min after two or fewer hours of continuous work on the computer (21).

### 2.5 Data quality assurance

The questionnaire was prepared in English, translated into Amharic after discussion with language experts, and retranslated back to English to check for consistency. Data collectors received training for 3 days on the objectives, procedures and techniques for data collection, as well as on familiarizing themselves with the study instrument. Participants were informed in detail on how to answer the questions. The questioners were checked for completeness and any illogical answers. Subsequently, the study questionnaire was pretested on 5% of the sample size of the study population to check for reliability, practicability, clarity, and whether the tool allows for the legible collection of the data, and then necessary modifications were made afterward.

### 2.6 Data management and analysis

Validation criteria were preliminarily set on Kobo Toolbox to limit the response range and avoid invalid answers. Subsequently, data was exported to SPSS version 26 for further analysis.

Descriptive statistics: frequency and proportion were used to analyze symptoms and prevalence of CVS. Results were presented with narration, tabulation, and graphical presentation.

Normality and outliers were checked. Multicollinearity among variables was assessed using the variance inflation factor (VIF) and corrected if any variables showed a VIF > 5.

Binary logistic regression analysis was performed for factors affecting CVS. Those explanatory variables with a *p* value less than 0.2 in binary logistic regression analysis were candidates for multiple logistic regression analysis. Variables with *p* < 0.05 and 95% confidence level in the multivariable analysis were considered statistically significant. An adjusted Odds Ratio (AOR) with a 95% confidence interval was used to report the strength of the association.

## 3 Results

### 3.1 Socio-demographic characteristics of study participants

A total of 497 employees of Ethio Telecom participated in the study over 5 weeks, from April to May 2023, with a response rate of 98.2%. Table 1 shows that just over half 270 (54.3%) were male. The median age (median interquartile range) of the participants was 32 (29–38).

**Table 1:** Socio-demographic characteristics of computer user Ethio telecom workers, 2023.

Variable	Category	Frequency	Percent
Sex	Male	270	54.3%
	Female	227	45.7%
Age	20–29 years	132	26.6%
	30–39 years	251	50.5%
	≥40 years	114	22.9%
Work experience	<6 years	214	43.1%
	≥6 years	283	56.9%

Regarding computer use, the majority of the participants (76.7%) reported that they used computers for more than 6 h per day. Additionally, Over half (56.9%) had six or more years of experience working with computers in their workplace.

### 3.2 Personal characteristics of study participants

As shown in Table 2, 143 (28.8%) participants reported using eyeglasses, and 48 (9.7%) reported a previous diagnosis of eye illness. Only 32 participants (6.4%) reported using lubricant eye drops while working on computers.

**Table 2:** Distribution of study participants according to personal characteristics of computer user Ethio telecom workers, 2023.

Variable	Category	Frequency	Percent
Eyeglass use	Yes	143	28.8%
	No	354	71.2%
Previous history of eye illness	Yes	48	9.7%
	No	449	90.3%
Lubricant eye drops use	Yes	32	6.4%
	No	465	93.6%
Awareness of the 20–20-20 rule	Yes	29	5.8%
	No	468	94.2%

Regarding awareness of CVS, 136 participants (27.6%) reported that they had heard about CVS, and substantially few participants (29, 5.8%) reported that they were familiar with the 20–20-20 rule.

### 3.3 Environmental and work-related characteristics

A substantial proportion of participants (88.3%) indicated that they had access to ergonomic adjustable chairs in their workplace. However, only 170 participants (34.2%) reported regularly taking breaks during their working hours. Notably, none of the participants used an anti-glare screen filter on their computers.

With regards to viewing distance, 25.8% of the participants sat in a way where the distance between their eyes and the top of the screen was below 50 cm. The majority of the participants, 360 (72.4%), reported that they adjusted their computers’ brightness and contrast to match the surrounding light levels. Most of the participants, 370 (74.4%) used laptops for their routine tasks at work. Local lighting was present in the majority 312 (62.8%) of the participants’ task area. Shows from the total study participants, 229 (46.1%), 145 (29.2%), and 121 (24.3%) sat aside, at the back, and in front of the windows, respectively.

### 3.4 Lighting assessment

**Figure 2:**
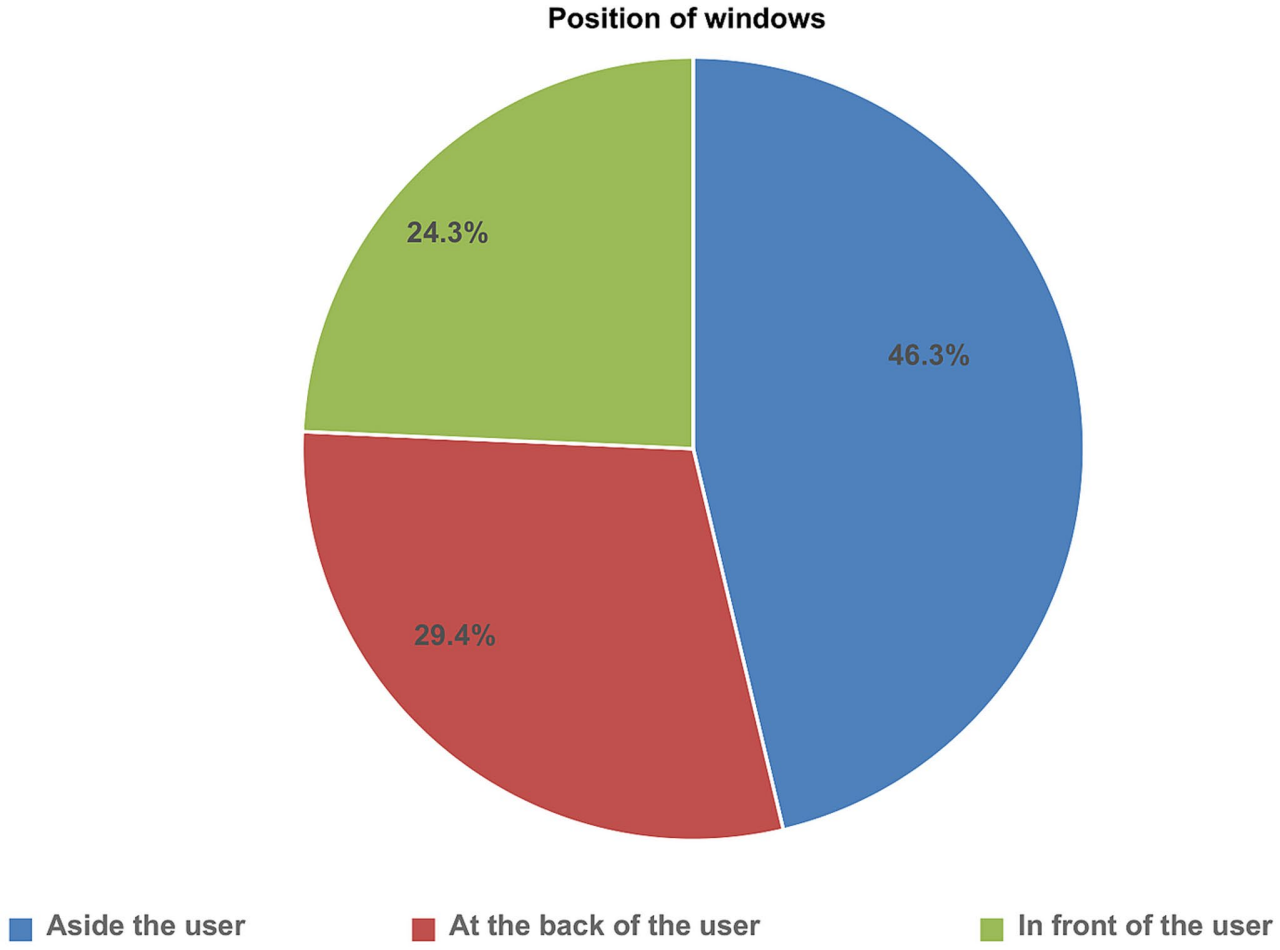
Distribution of study participants’ sitting position to the position of windows of computer user Ethio telecom workers, 2023.

Just over half of the study participants, 276 (55.5%), worked under lighting of ≥200 LUX. 221 (44.5%) of the participants worked under lighting of ≤200 LUX Figure 2.

### 3.5 Prevalence of CVS

**Figure 3:**
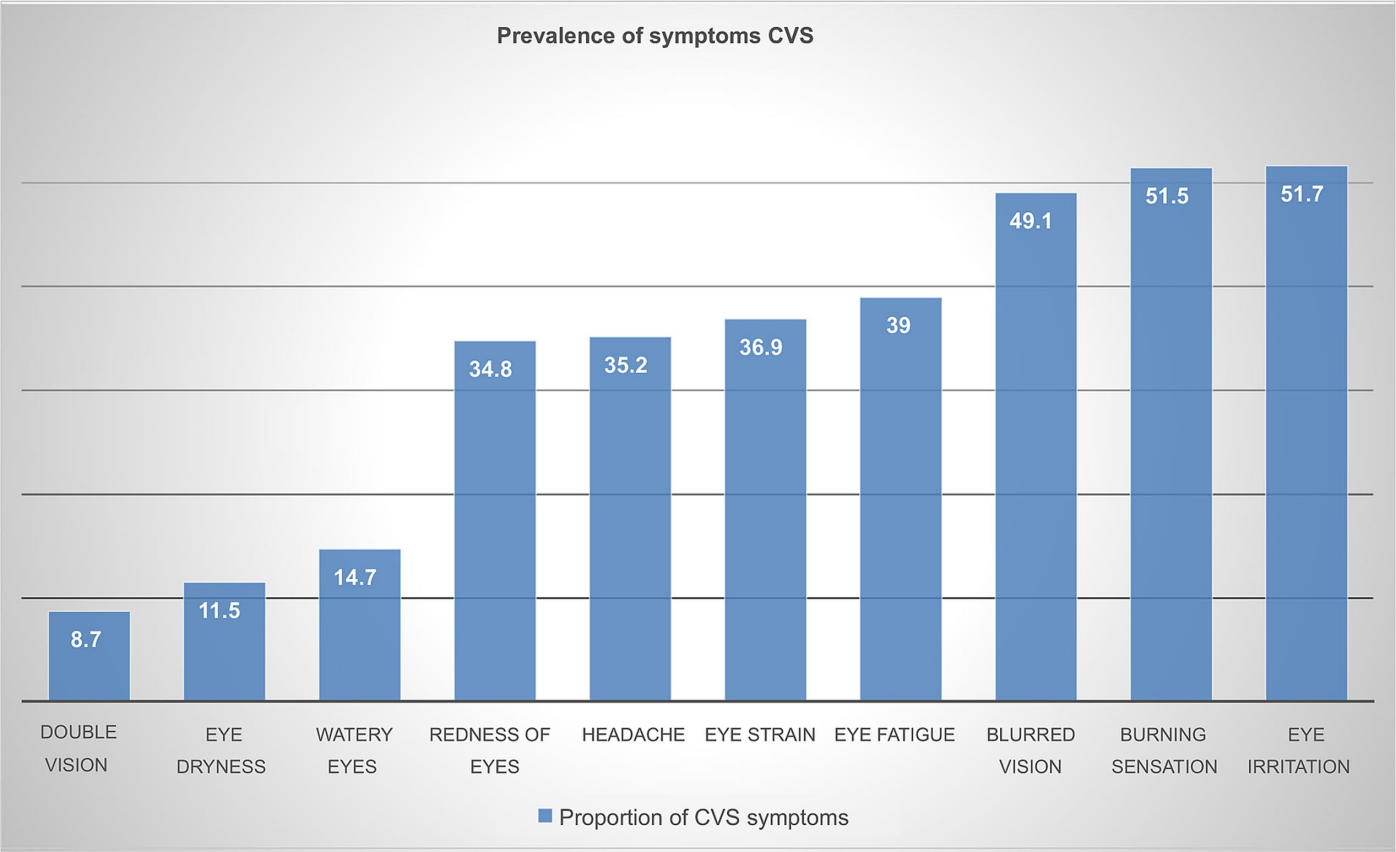
Prevalence of symptoms among computer user Ethio telecom workers in Addis Ababa, 2023.

The prevalence of CVS among Ethio telecom zonal office workers during the last 12 months was 68.8% [95% CI (64.5, 72.9)]. As depicted in Figure 3. the most commonly reported symptoms were eye irritation (51.7%), burning sensation (51.5%), and blurred vision (49.1%). Whereas, double vision (8.7%) and eye dryness (11.5%) were reported the least. There is no significant difference in the prevalence of CVS between males 184 (54%) and females 154 (46%), *p*-value>0.05.

In the multivariate logistic regression factors such as the habit of taking breaks, adjustment of brightness and contrast, viewing distance, and task illumination showed a significant association at below 0.05 *p*-value as can be seen in Table 3.

**Table 3:** Multivariate analysis for computer vision syndrome among computer user Ethio telecom workers in Addis Ababa.

Variables	Categories	Computer vision syndrome		COR (95% CI)	AOR (95% CI)	*p*-value
		Yes	No			
Age	20–29	73	59	1	1	
	30–39	163	83	2.009 (1.296–3.116)	1.617 (0.965–2.708)	0.068
	>40	252	131	3.031 (1.721–5.338)	2.016 (0.994–4.092)	0.052
Years of work on computer	<6 years	123	78	1	1	
	≥ 6 years	207	77	1.594 (1.087–2.336)	0.941 (0.579–1.530)	0.476
Working hours per day	<6 h	230	124	1	1	
	>6 h	112	31	1.594 (1.087–2.336)	1.565 (0.714–1.902)	0.808
Habit of taking breaks	No	255	72	1	1	
	Yes	87	83	0.29 (0.199–0.441)	0.439 (0.281–0.686)	<0.0001
Eyeglass use	No	230	124	1	1	
	Yes	112	31	1.948 (1.237–3.066)	1.566 (0.922–2.658)	0.095
Pre-existing eye illness	No	302	147	1	1	
	Yes	40	8	2.434 (1.111–5.332)	1.637 (0.663–4.037)	0.095
Adjustment of brightness and contrast	No	118	19	1	1	
	Yes	224	136	0.265 (0.156–0.450)	0.389 (0.221–0.684)	0.01
Blinds and Curtains	No	103	28	1	1	
	Yes	239	127	0.512 (0.320–0.818)	0.727 (0.420–1.258)	0.254
Ergonomic adjustable chair	No	144	11	1	1	
	Yes	295	47	0.479 (0.241–0.952)	0.763 (0.349–1.670)	0.498
Viewing distance	≥50 cm	230	139	1	1	
	<50 cm	112	16	4.23 (2.405–7.440)	2.315 (1.238–4.330)	0.009
Task illumination	Proper	160	116	1	1	
	Improper	182	39	1.383 (2.222–5.151)	1.782 (1.090–2.914)	0.021

1, reference; COR, Crude odds ratio; AOR, Adjusted odds ratio; CI, confidence interval.

In this study, individuals with a habit of taking breaks were 56% (AOR: 0.44, 95% CI (0.28–0.69)) less likely to develop CVS as compared to those individuals who did not have a habit of taking breaks. Similarly, individuals from this study who adjust the brightness and contrast of their computer screen were 61% (AOR: 0.39, 95% CI (0.22–0.68)) less likely to develop CVS when compared to those individuals who do not adjust the brightness and contrast of their computer screens.

While distance between participants and their computer screen (viewing distance) was significantly associated with CVS. Employees who viewed their computer screens at distances below 50 cm, were 2.3 times likely to develop CVS as compared to those employees who viewed their computer screens from a distance more than 50 cm, (AOR 2.32, 95% CI (1.24–4.33)).

Workers working under improper task illumination (<200 LUX) were 1.8 times more likely to develop CVS when compared to individuals working under proper illumination conditions (≥200 LUX), (AOR: 1.78 95% CI (1.09–2.91)).

## 4 Discussion

In this study, the prevalence of CVS among employees of Ethio telecom zonal workers was 68.8% [95% CI (64.57–72.9)]. Factors significantly associated with CVS included the habit of taking breaks, adjustment of brightness and contrast, viewing distance <50 cm and improper task illumination.

The prevalence of CVS in this study was closely aligned with other studies: 67.4% among computer office workers in Sri Lanka (7), 72% among computer engineering students in Pakistan (8), 70.4 among university instructors in Ethiopia (14), and 69.5% of computer users in Ethiopia (15). The similarities in this estimates may be attributed to the use of a comparable definition of CVS symptoms.

However, higher prevalences have been reported elsewhere: 89.9% in Malaysia (2), 94.5% in Jordan (16), and 81.0% in Thailand (22). The possible reason for the higher prevalence reported could be that study participants are students the amount of time spent on computers could increase significantly and the habit of taking breaks could be limited as they are more focused on their studies and exams or that they may not have a fixed reading and studying places as they may use any places to study they may study in their dormitories where lighting conditions may not be as appropriate.

Then again, the differences in the tools used to assess CVS and the definition of CVS might be the reason for the variation in prevalence estimates. For example, the study in Jordan used a 16-symptom questionnaire to determine the presence of CVS. In contrast to this, this study used a 10 symptoms questionnaire.

In this study, eye irritation and burning sensation were the most prevalent symptoms reported this is in line with a study among bank workers in Ethiopia (6), and the similarity may be attributed to the comparable exposure status of employees having similar workstations and the similarities in demographics with these working groups.

Blurred vision was another prevalent symptom, reported by 49.1% of the participants. Similarly, it was reported by 45.7% of individuals in a university in Jordan (16). This similarity could be attributed to blurred vision being associated with several other conditions in individuals such as sight problems, astigmatism, and other underlying medical conditions. There is a similarity in the experiences of these two groups where over half of the participants worked with digital devices for at least 4 years or more. This prolonged exposure could perhaps explain the similarities in symptoms (9).

Several studies suggest that females are more likely to develop CVS than males perhaps due to hormonal level differences between female and male as well as female hormonal cycles which impacts evaporation from the tear film (7, 22, 23). Whereas, few other studies show a higher proportion of males developing CVS (6, 10). In this study, however, we did not find a significant difference between males and females.

A substantial proportion of participants in our study were aged below 40. Age was not significantly associated with CVS in this study. However, age is perhaps a known predictor of vision-related problems. As people age, they start to have difficulties of seeing clearly at closer objects, particularly when working with computers. There is a link between chronic conditions such as diabetes and vision; as people get older, they are at risk of developing these conditions, which in turn lead to vision related problems. This relationship is also documented in other studies (24).

In multivariable regression analysis, habits of taking rest breaks was a significant predictor of CVS among participants. Those who regularly took rest breaks were 56.1% less likely to develop CVS compared to those who did not. This finding is in line with other studies showing a similar pattern of decreased risk when taking rest breaks (5, 6, 11, 12, 25). This is perhaps due to increased blinking rates while taking breaks as reduced blinking rates are considered often to lead to dry and irritated eyes (26). In many instances, it is suggested to take micro-breaks during tasks that require sitting in the same posture (13). And the importance of the 20–20-20 rule could never be stressed enough. The American Optometric Society (AOA) suggests adhering to the 20–20-20 rule, which states that you should spend at least 20 s every 20 min gazing at something that is 20 feet away. This enables your eyes to unwind and lubricate more due to greater blinking. Taking frequent pauses can help individuals focus better (1).

In this study, it is seen that participants adjusting brightness and contrast were 61.1% less likely to develop CVS compared to those who did not adjust their brightness. This finding is supported by other similar studies (18, 27). It is suggested to keep screen brightness to match the ambient light. Some modern devices often adjust brightness automatically to match the ambient light using sensors but it is not possible to always rely on this as most devices in an office setting may not have this feature. As for contrast it is often recommended to increase contrast to make the texts that are read outstand the background and hence reduce eyestrain.

In this study, we found a significant association between viewing distance and the development of CVS. Participants who watched their computers from a distance of less than 50 cm were 2.3 times more likely to develop CVS than those who viewed them from a distance of more than 50 cm. There are studies in agreement with this finding (28, 29). The possible reason for this again has to do with the reduced blinking rate when sitting too close to monitors. Between 20 and 40 inches is the optimal distance from your eyes to the screen. The ideal distance for this is roughly an arm’s length. Based on the size and resolution of the screen, you can also determine the appropriate distance. You can sit back in the proper posture without slouching too far back or forward if you have the proper distance between your monitor and your eyes (30).

It was also reported in this study when compared to people who worked under sufficient illumination circumstances (≥200), workers who were working under inadequate task illumination (<200 LUX) had a 1.8 times higher risk of developing CVS. Some studies support this finding (1, 5, 31). Although there is no clear-cut value for the level of illumination, it is recommended to keep office workplaces well-illuminated and not below 200 LUX (19). As described in many instances working under poor lighting conditions can cause eye strain and dry eyes. Perhaps, this is possibly due to the extra load put on the eyes to focus more during poor lighting conditions and as with any other muscles of the body the muscles of the eyes get overworked and tired.

## 5 Conclusion

This study aimed to assess the magnitude of Computer Vision Syndrome and identify its possible predictors among Ethio Telecom computer using workers. Consequently, this study reported the prevalence of CVS among employees of Ethio telecom zonal office workers in Addis Ababa was 68.8% [95% CI (64.5, 72.9)]. Moreover, this study identified that habits of taking rest breaks, adjustment of computer brightness and contrast, viewing distance and task illumination levels were significantly associated to CVS.

Limitations in this study arise from the design. Possibly information bias could be introduced such as recall bias where participants might not readily remember symptoms for the major outcome and other variables of the study and Hawthorne effect could also come into play where participants are aware that they are being observed and act in unusual manner. However, steps were taken to minimize this such as explanation of the questions in simple language and allocating sufficient time for responses. In addition, sufficient training was provided to data collectors on the proper use of instruments and data collection.

Recent evidences suggest a link between built environment and visual comfort particularly room design (32). This study did not extensively cover such factors. Future research could investigate the links of CVS and built environment with a focus on room size, design, and lighting conditions besides intensity such as light color and temperature.

## Data Availability

The raw data supporting the conclusions of this article will be made available by the authors, without undue reservation.
